# Evaluation of the tuberculosis programme in Ningxia Hui Autonomous region, the People’s Republic of China: a retrospective case study

**DOI:** 10.1186/1471-2458-12-1110

**Published:** 2012-12-23

**Authors:** Yu Rong Yang, Donald P McManus, Darren J Gray, Xiao Ling Wang, Shu Kun Yang, Allen G Ross, Gail M Williams, Magda K Ellis

**Affiliations:** 1 Ningxia Medical University, Ningxia Hui Autonomous Region, Ningxia, the People’s Republic of China; 2 Molecular Parasitology Laboratory, Queensland Institute of Medical Research, Brisbane, Australia; 3 School of Population Health, University of Queensland, Brisbane, Australia; 4 Ningxia Infectious Disease Hospital, Yinchuan, Ningxia Hui Autonomous Region, Yinchuan, People’s Republic of China; 5 The first Yinchuan City Hospital, Yinchuan, Ningxia Hui Autonomous Region, Ningxia, People’s Republic of China; 6 Griffith Health Institute, Griffith University, Brisbane, Australia; 7 Wellcome Trust Centre for Human Genetics, Oxford University, Oxford, UK; 8 Centenary Institute, Sydney, Australia

**Keywords:** Prevention and control, Communicable disease control, Directly observed treatment short course strategy, DOTS

## Abstract

**Background:**

Tuberculosis is a devastating disease due to its rapid transmission and high rate of mortality. Ningxia Hui Autonomous Region (NHAR), located in the North-west, is one of the poorest provinces in China and national surveys have shown TB has been hyper endemic in NHAR for several decades. As no active surveys had been undertaken since the initiation of the DOTS control program across all of NHAR.

**Methods:**

A retrospective study was undertaken of all clinical records of TB patients registered from January 2005 to September 2009. Poisson regression was performed to investigate the change in incidence over time and accounted for age, sex and county. Length of time on treatment, disease severity and patient delay were assessed by county.

**Results:**

More than 30% of patients had been on treatment for over 12 months and 10% for over 3 years, reflecting drug-resistance or failure of DOTS. More than 93% of patients had grade III disease at time of diagnosis and >15% of patients had severe disease grade IV-V in some NHAR counties. Further, 8.8% of patients were not diagnosed for over 6 months from the onset of symptoms; this was as high as 20% in some counties. The reported incidence of TB is most likely grossly underestimated and the data indicate TB is a major public health concern in NHAR.

**Conclusions:**

It is clear that active surveillance is necessary to determine the full extent of the burden of TB in NHAR. New control and treatment strategies for TB are required that increase awareness in the health-care system and at the individual and community level.

## Background

The Ministry of Health of the People’s Republic of China (PRC) estimates that 5 million people currently have active TB with 80% of cases in rural areas, particularly in the poorest provinces in north and north-western PRC - including Ningxia Hui Autonomous Region (NHAR) [1].

Four national TB surveys have been undertaken in PRC in 1979, 1985, 1990 and 2000. The first reported a high prevalence of 885 cases/100,000 population with a low sputum smear-positive (SSP) detection rate (121/100,000). The prevalence had decreased to 536/100,000 in 1990 reflecting an average annual decrease in prevalence of 4.5%. Meanwhile, the SSP rate had increased by 3.0% per annum to 168/100,000 [2]. The prevalence continued to decrease slowly during the 1990s (3.2% per annum) to 367/100,000 in 2000 [3,4].

According to World Health Organization reports, the prevalence for PRC in 2000 fell from 214 to 154 per 100,000 from 2000 to 2006; an average of 5.6% per year [5]. The decrease slowed during the late 2000s, decreasing only 5.6% over the following 3 years. Incidence remained relatively stable during this time, decreasing by < 1% per annum over the course of the last decade. This trend reflects the change in SSP rate of new cases which had increased in PRC from 34% in 2000 to 75% in 2008.

It is clear from the National survey reports for PRC that NHAR has been hyper-endemic for TB for many decades. The reported prevalence in 1979 showed NHAR had the fourth highest in the country with an estimated 1003 cases/100,000 population. Prevalence had dropped substantially by 1990 to 530/100,000, a figure below the national average. This dramatic reduction in prevalence not only in NHAR but across many parts of PRC could reflect, in part, a change in diagnostic practices for smear-negative patients based on chest X-ray (CXR) anomalies thus reducing the number of false-positive diagnoses. Despite this, the prevalence had rebounded to 619/100,000 in 2000, placing NHAR as the third most TB prevalent region in PRC. Only following the SARS outbreak in 2002/2003 [6] was the nationwide internet-based communicable disease reporting system introduced as part of the disease surveillance system for PRC [7] and the DOTS program instigated country-wide. By the end of 2004, 93% of the county and township levels in NHAR were covered by the program, comparable to levels in other parts of the PRC [8].

Despite the evident long-standing TB endemicity, there is very limited published literature for NHAR, with the exception of a small number of local government documents and short journal reports. As no active surveys had been undertaken since the initiation of the DOTS control program in NHAR, we systematically collected hospital record data for the period January 2005 to September 2009 so as to provide essential information requisite for the design and implementation of future TB control strategies.

## Methods

### Study area

**Figure 1:**
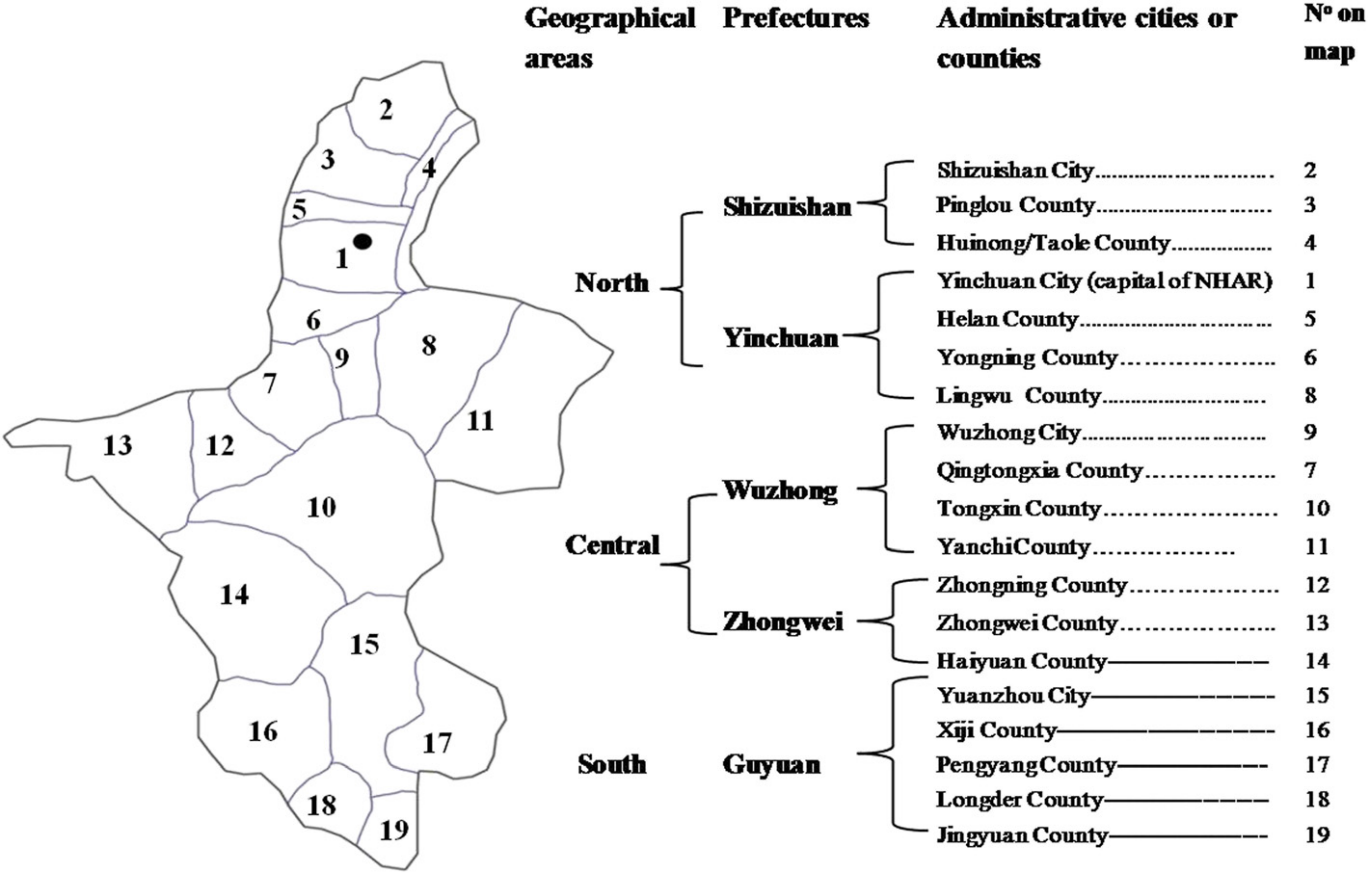
Map of Ningxia Hui Autonomous Region, People’s Republic of China.

NHAR has a population of around 5.6 million and is the smallest provincial autonomous region on the Loess plateau in northwest China with a total area of 66,400 square km. NHAR is divided into 5 prefectures, located in three natural geographic zones (Figure 1). The regional disparity in economic development is characterised by the lowest levels of gross domestic product (GDP) in the south and central areas, with the highest in urban Yinchuan and the surrounding counties in the north [9].

### Data collection

Patients diagnosed with TB between January 1, 2005 and September 30, 2009 were reported to the Division of Tuberculosis Control of the Department of Public Health of NHAR. This provincial level electronic reporting system was introduced in NHAR at the beginning of 2005. Routine demographic data collected at the time of diagnosis for each case included: age, sex, and domicile. Specific information concerning TB included: the date of onset of symptoms; date of diagnosis; clinical site/s of disease; results of microbiology tests (sputum smears and cultures); treatment regime, and treatment outcome.

### Case management

Diagnosis of pulmonary TB in hospitals and TB clinics is made on the basis of clinical examination; chest radiography and sputum smear microscopy and/or sputum culture. Following diagnosis, patients enter the DOTS program which prescribes short-course chemotherapy (SCC) comprising 2 months of isoniazid (H), rifampicin (R), pyrazinamide (Z) plus streptomycin (S) or ethambutol (E) followed by 4 months of H and R. This is the WHO recommended regimen for treating new cases of smear-positive pulmonary TB or smear-negative pulmonary TB with substantial radiographic evidence of active disease [10]. For re-treatment of patients who have had previous therapy (relapse or resumption of treatment after interruption), an 8-month regimen has been recommended by the WHO [10]. During the initial intensive phase of treatment, patients are supervised by health workers. Following this, the program is continued at the family/community level. This system facilitates the administration and re-prescription of medication for patients in the DOTS program. The TB treatment strategy in NHAR (of which 85% is rural) is predominantly community-based (CB), involving directly observed treatment with CB-DOTS.

### Statistical analysis

Data analyses were performed using Microsoft Excel, EPI-INFO (Centres of Disease Control and Prevention, Atlanta) and SAS (SAS Institute, Inc, Cary, NC). Direct age standardised incidence (per 100,000 population) were calculated using public health records and the 2006 NHAR census provided by the National Statistical Service of China [11]. Poisson regression was performed using GENMOD in SAS (SAS Institute, Inc, Cary, NC) and accounted for age, sex and county. The number of individuals on treatment for more than 1 year were calculated as the number of new cases registered prior to 1^st^ October 2008 who had taken more than 1 year to complete treatment or who were still taking treatment on this date. A cut-off date of 1 October 2007 was applied when calculating those who had been on treatment for >2 years. Time to diagnosis was calculated as the number of days from the date of onset of symptoms to the date of diagnosis. The date of onset of symptoms was defined as the first occurrence of the main complaint and symptom related to TB infection. This was further broken down by days from onset of symptoms to seeking medical treatment and number of days from seeking medical treatment to diagnosis. The case status of patients in Yongning County was coded differently to other counties in the province and, as such, this county was omitted from all new case data analyses.

### Ethical considerations

The study was approved by the Research and Ethics Committee of Ningxia Medical University, Yinchuan, P.R. China, and official letters requesting authorisation for retrospective data collections were signed by the Head of this University. Letters were sent to all TB coordinators of TB referral hospitals and clinics in NHAR involved in the study. The letters introduced the nature and scope of the research to the coordinators throughout NHAR so that permission was obtained for the study to be undertaken.

## Results

### TB incidence by year and location

**Figure 2:**
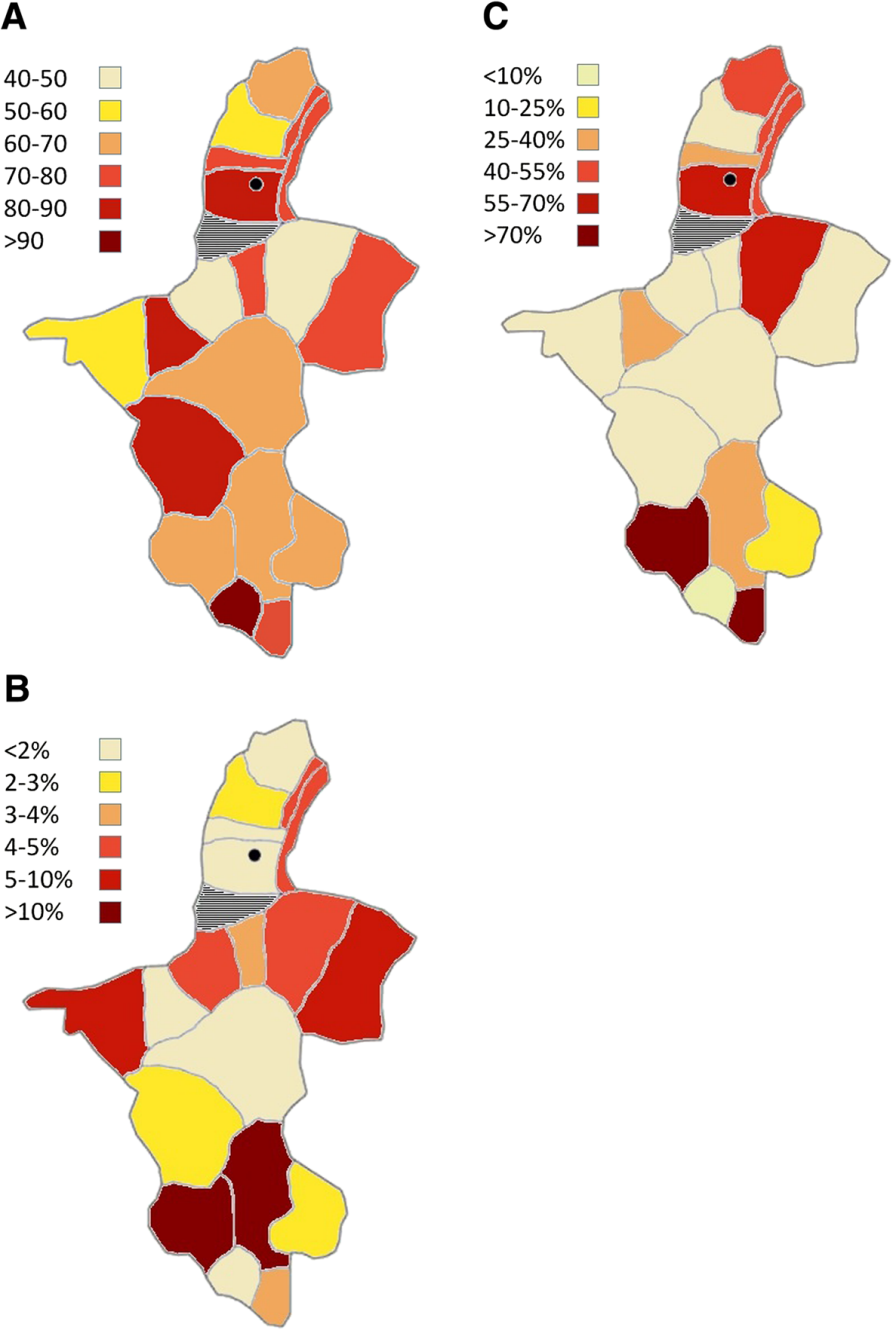
**Distribution of disease and infection indicators by county: (A)Shows the average TB incidence (per 100,000 population); (B) Shows the proportion of advanced stage cases in 2009; (C) Shows the average percentage of cases continuing treatment > 1 year.**

As data for 2009 were only collected from January 1-September 30, incidence was calculated for the period 2005 – 2008. These data are presented in Table 1 and show the incidence in the different administrative districts or counties by year for NHAR. The average incidence over the 4-year period is shown by county in Figure 2A.

**Table 1:** The notification rate of human TB cases from 2005–2008 in NHAR by county

**Cases by year County (population/km**^**2**^**)**	**2005**	**2006**	**2007**	**2008**	**2005-08**
	**Incidence (Cases)**	**Incidence (Cases)**	**Incidence (Cases)**	**Incidence (Cases)**	**Average Incidence (95% CI)**
Longde (220)	92.6	144.2	125.5	111.4	117.9 (102.1-133.7)
Yinchuan (271)	65.9	79.9	103.5	94.3	84.4 (72.8-96.0)
Zhongning (77)	86.6	95.8	82.0	80.2	83.0 (62.3-103.6)
Haiyuan (54)	63.2	116.0	76.2	81.4	82.4 (68.3-96.5)
Jingyuan (149)	57.7	84.6	97.8	89.6	79.9 (68.3-91.6)
Wuzhong (269)	63.4	89.3	84.7	84.6	79.9 (64.7-95.0)
Helan (141)	73.5	75.7	86.1	86.9	77.1 (59.6-94.7)
Huinong + Taole (73)	62.4	86.4	51.7	105.3	75.0 (56.6-93.3)
Yanchi (21)	72.2	63.5	82.4	71.9	70.0 (55.7-84.4)
Tongxin (42)	61.6	72.3	79.6	68.4	68.4 (56.6-80.1)
Xiji (144)	56.2	82.4	59.2	74.1	67.5 (60.0-74.9)
Shizuishan (150)	64.3	74.9	74.4	66.1	67.0 (54.7-79.3)
Guyuan (132)	55.2	69.7	69.3	59.0	61.8 (54.9-68.8)
Pengyang (98)	43.7	86.3	60.9	58.7	61.8 (53.1-70.4)
Pinglou (108)	55.8	58.6	52.8	54.2	53.2 (40.8-65.5)
Zhongwei (71)	43.8	59.4	55.9	66.6	53.1 (39.3-67.0)
Lingwu (70)	49.1	46.8	44.5	55.4	48.0 (38.0-57.9)
Qingtongxia (128)	49.9	49.9	50.9	43.7	46.1 (35.0-57.2)
Total Ningxia	62.0 (2566)	80.5 (3405)	75.0 (3093)	75.5 (3140)	70.1 (66.9-73.2)

During this 4-year period, the greatest change in incidence was evident between 2005 and 2006. This is due to the striking increase in incidence in the counties in the southern prefecture which collectively showed an average increase of 55.7%. This was followed by the Zhongwei prefecture which had an average 43% increase in incidence. The incidence appeared to decrease in all counties in 2007 with the exception of Jingyuan, which continued to increase by a further 16% until subsiding in 2008. While the annual incidence from 2006 to 2008 appeared to remain reasonably constant throughout this 3-year period, a number of counties appeared to experience some fluctuations in incidence rates. In particular, the counties of Yinchuan, Jingyuan, Tongxin and Yanchi had peak incidence levels in 2007, which subsided in 2008 and while the majority of all counties had decreased incidence rates in 2008 compared with 2007, the county of ‘Huinong and Taole’ increased sharply by 104%. Longde county had the highest reported number of TB cases in the province for each year from 2005 to 2008, followed by the urban region of Yinchuan city. The lowest incidence rates observed throughout the 4-year period were observed in the central districts of Qingtongxia and Lingwu and in the northern county of Pinglou. Poisson regression showed that across NHAR as a whole, there was a significant increase in TB incidence from 2005 to 2008 as reflected by an overall relative risk of 1.044 (95% CI 1.027-1.061; P < 000001).

### TB incidence by age and sex

**Figure 3:**
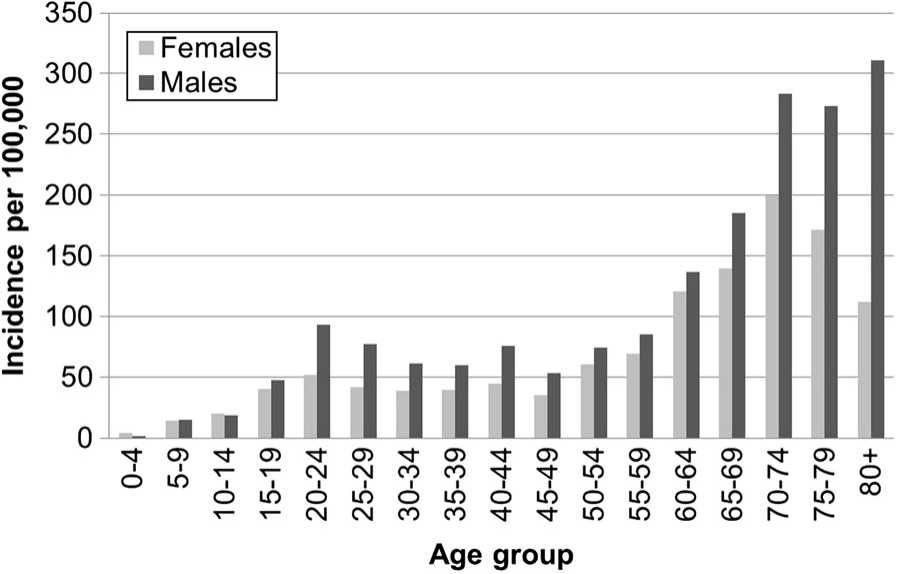
The distribution of TB incidence by age in males and females.

The distribution of new TB cases in males and females in different age groups (based on the 2006 population census) [11] is shown in Figure 3. The lowest incidence occurred in the youngest age groups between 0 and 19 years of age with a small peak in young adults (20–25 years of age) particularly in males. In general, the incidence was higher in males than in females. This was more markedly observed in subjects aged over 65.

### Clinical features of NHAR TB cases

The majority of TB patients were found to be new cases, accounting for 94% of the entire data set. The reporting system introduced in 2005 registered all TB cases as new incident cases without consideration of prior TB diagnosis so that the proportion of previously treated cases may have been underestimated. The proportion of new cases, however, was found to be similar each year. Of the TB cases, 97% (16165/16601) were pulmonary, 2.8% (365/16601) were pleural, and the remainder were extra-pulmonary. The low proportion of extra-pulmonary TB in this study is also likely to be an underestimation due to most extra-pulmonary TB cases being registered at general hospitals rather than TB dispensaries given the more severe nature of the disease in most cases. Amongst the pulmonary TB cases, 67% (11117/16165) were sputum-positive at the time of diagnosis. Based on X-ray analysis (TB diagnosis guidelines) [12], TB grading of patients was categorized into five types: early stage grades I and II; mid-stage grade III; and later stage cases, grades IV and V.

The most commonly observed stage amongst all new cases was grade III (93.1% across all counties). The number of advanced stage new cases slowly decreased from 2006–2008 but rebounded rapidly again in 2009 to 5.2% of new cases. Further investigation of these data at the county level showed that Xiji and Guyuan counties accounted for most of these cases with prevalences of 17.03% and 18.95%, respectively (Figure 2B). Longde and Tongxin counties reported the lowest number of advanced stage cases throughout the 5-year period (an average of 0.24% and 0.19%, respectively).

### Treatment of TB

We investigated the length of time of treatment in all new registered cases. Thirty percent of all new cases were found to have been continuing treatment for longer than one year and 42% of all new cases diagnosed before 30^th^ September 2007 were found to have continuing treatment for longer than 2 years. Investigation of these cases by county indicated the distribution to be random with the majority of counties having less than 15% of patients on treatment for over 12 months (Figure 2C). The county with the highest rate of long-term treatment of patients was Xiji in south NHAR with 84.3% receiving treatment for over a year. There appeared to be no overlap between the distribution of long-term treatment cases (Figure 2B), and either disease severity (Figure 2C) or incidence (Figure 2A).

### Incidence and time to diagnosis

**Figure 4:**
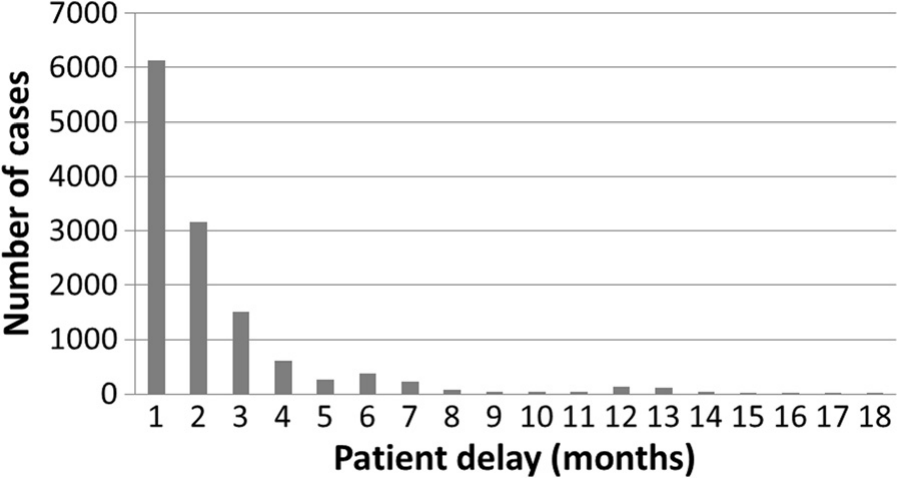
The distribution of patient delay (data shown only up to 18 months).

The time from the onset of symptoms to that of diagnosis and subsequent treatment is important as this period reflects the time of infectiousness of a patient. These indices are shown for the different counties in Table 2. Between 2005 and 2009, 8.8% of all new cases remained undiagnosed for six months. Overall, 74.2% of cases were found to have sought treatment within the first three months from the onset of symptoms (Figure 4). However, the time taken to seek treatment varied between counties. More than 20% of patients in Yanchi, Pengyang and Jinchuan and over 15% in Longde and Wuzhong had waited longer than six months to seek medical treatment following the onset of symptoms. Shizuishan had the highest proportion of new cases (6.1%) who had sought medical attention but who had waited over 90 days to be correctly diagnosed.

**Table 2:** Time from onset-of-symptoms to seeking medical attention and to diagnosis of new cases by county*

**County**	**N (total)**	**Days to seek treatment (range)**	**N > 6 months (proportion)**	**Days to diagnosis (range)**	**N > 6 months (proportion)**
Yinchuan	2209 (2359)	3 (0–1606)	38 (2.2%)	8 (0–2106)	45 (2.61%)
Shizuishan	314 (322)	17 (0–802)	16 (5.1%)	33 (0–1096)	25 (7.96%)
Pinglou	523 (528)	19 (0–221)	1 (0 · 2%)	22 (0–354)	3 (0 · 6%)
Huinong	260 (485)	24 (0–296)	3 (1 · 2%)	30 (0–461)	5 (1 · 9%)
Helan	544 (570)	33 (0–2616)	62 (11.4%)	36 (0–3765)	67 (12.3%)
Qingtongxia	427 (434)	25 (0–1479)	5 (1.2%)	34 (0–2822)	20 (4.7%)
Lingwu	468 (480)	28 (0–980)	9 (1 9%)	35 (0–319)	14 (3.0%)
Wuzhong	706 (711)	42 (0–2160)	115 (16.3%)	62 (0–2693)	139 (19.7%)
Tongxin	1031 (1031)	31 (0–1407)	51 (5.0%)	33 (0–420)	54 (5 · 2%)
Yanchi	390 (391)	76 (0–2201)	94 (24 · 1%)	84 (0–2339)	100 (25.6%)
Zhongning	725 (730)	34 (0–797)	39 (5.4%)	38 (0–4831)	48 (6.6%)
Zhongwei	705 (705)	36 (0–1841)	53 (7 · 5%)	47 (0–2170)	62 (8.8%)
Haiyuan	1309 (1310)	31 (0–1405)	49 (3.7%)	33 (0–4320)	53 (4.1%)
Guyuan	906 (1356)	63 (0–2005)	99 (10.9%)	65 (0–2352)	100 (11.0%)
Xiji	1115 (1432)	2 (0–426)	43 (3.9%)	4 (0–704)	43 (3.9%)
Pengyang	637 (653)	64 (0–2610)	129 (20.3%)	68 (0–3153)	140 (22.0%)
Longde	834 (838)	64 (0–1733)	132 (15.8%)	66 (0–1483)	145 (17.4%)
Jingyuan	345 (363)	63 (0–1098)	76 (22.0%)	70 (0–1501)	82 (23.8%)

*included cases diagnosed between 1^st^ January to September 30^th^ (case records were unavailable for October to December) · † All times are shown as median number of days; N > 6 months indicates the number of individuals who took longer than 6 months to seek treatment/be diagnosed.

## Discussion

National surveys undertaken in the PRC from 1979–90 showed that although the prevalence of TB fell by an average of 3.3% annually during the 1980s, the notification rate represented only 30% of the total burden in the 1990s [13]. This was due mainly to the collapse of the public health system which particularly affected rural communities throughout the PRC [14]. The reduction in government expenditure in the health sector fell from 32% in 1972 to 16% in 2002. This forced many Chinese health-care facilities and providers to run as businesses, requiring patients to pay for their diagnostic tests and treatment regimens. Many patients could not afford to maintain consistent and regular treatment for the required period due to the high cost of anti-TB drugs, which may reflect the rapid increase in the prevalence of multidrug-resistant (MDR) TB [15]. This inadequate TB control thus reflected the heavily malfunctioning health system during this period in the PRC.

TB has been particularly hyper-endemic in the underdeveloped areas in north-western PRC for several decades. The high-prevalence in NHAR as reported in the 2000 national survey placed it as the third most prevalent autonomous region/province in PRC and 1.67 times higher than the national average (619/100,000 vs. 367/100,000). The prevalence in PRC then was 4.6 times higher than the global average prevalence for TB of 139/100,000.

Scrutiny of the data presented here indicates the average incidence in NHAR from 2005–2008 was low (61/100,000) but it is likely that the reported cases substantially underestimated the total number of patients with TB as many individuals could have remained undiagnosed. The overall distribution of high incidence counties appears to be random and could be due to different TB transmission factors occurring in these areas. For example, Longde county in south NHAR had the highest proportion of individuals waiting over six months from the onset of symptoms to seeking medical advice (15.8%) which, combined with the highest population density in NHAR and low economic level, may account for the highest recorded incidence. In contrast, Haiyuan county had some of the highest incidence rates over the 4 year period, but has one of the lowest population densities in the province suggesting that specific geographical and/or socio-economic factors may contribute to TB transmission in this county. Although the Yinchuan urban Counties have higher per capita GDP levels [9], and thus would be expected to have a lower incidence of TB, they are impacted by the influx of seasonal migrants coming from more rural parts of NHAR during winter months to seek temporary employment. Further work is required to investigate the full effect that these migrants have on TB prevalence. The overall epidemic trend of notification rates in NHAR showed a gradual increase over the 4-year period. These rates appear to have fluctuated over this time with different trends observed in different counties; however, increased notification rates were observed in most counties overall. Such increased notification rates may suggest a higher rate of case identification rather than increased transmission. Similarly, several counties reported relatively low notification rates of TB compared to NHAR overall. While this could reflect lower transmission, it may equally indicate lower diagnosis rates. As this study focused on registered patient records only, subjects who failed to seek medical treatment or who were misdiagnosed, would have been missed. Accordingly, the numbers reported here likely underestimate the true disease burden attributable to TB in NHAR. Such under-reporting is an important issue since undisclosed cases will continue to spread the disease and thus may reflect the overall increase in notification rate during the 2005–2008 study period. Active screening should be undertaken to determine the true prevalence and incidence of TB in NHAR.

The low TB incidence evident in the very young (0–9 years of age) in NHAR reflects the difficulty of establishing a definitive diagnosis in this age group [16]. Interestingly, there was a small peak observed in young adult age (20–24 years of age) which may reflect high transmission rates. The higher incidence observed in some adult age categories could also reflect the different socio-economic status of these groups, as well as age-related immunological factors [17]. The results of this survey support previous studies that showed significantly higher TB incidence in males than females, which may reflect a genuine sex difference in TB susceptibility [18,19], or behavioural differences [20].

The length of time that patients took to seek medical attention (patient delay) and the delay in diagnosis is of concern in NHAR. More than 25% of all cases wait over three months to seek medical attention and 30% of these wait more than 6 months. Such patient delay was found to be particularly high in specific counties. The reason for patient delay in these counties is unknown but may reflect a lack of TB awareness and/or socio-economic considerations such as the cost of initial diagnosis and the distance patients needed to travel for medical attention. Although DOTS is free of charge in NHAR, it is only implemented once a clear diagnosis has been made. These factors are critical issues for the control of TB in NHAR. The lack of treatment increases morbidity and mortality at the patient level; this also increases exposure at the community level as patients remain infectious as long as they are untreated.

The prolonged time of treatment-to-cure observed in this study likely reflects failure of the DOTS program at the community level. As of the end of this study period, a total of 5394 patients were recorded as currently on treatment and, of these 58% had been receiving treatment for over two years. Many factors may contribute to this, including the difficulty in surveillance, due to large distances between patient residence and TB clinic, and chemotherapy non-compliance. Inadequate TB awareness in the community coupled with poor knowledge and/or training of local health workers may increase community exposure and fuel TB transmission. Thus evaluation of these factors should be a priority in order to develop new control strategies and identify early-stage cases for successful treatment by the DOTS program and minimise the transmission of infection.

## Conclusions

It is clear that NHAR has been hyper endemic for TB for several decades. The reported incidence of TB here is most likely grossly underestimated and the data indicate TB is a major public health concern in NHAR. Patient delay and poor compliance may reflect poor TB awareness in the community. Given the high proportion of patients on prolonged treatment, drug sensitivity testing is urgently required to assess the extent of drug resistance in NHAR. Further work is also required in NHAR to more fully understand the social and environmental determinants that contribute to the high transmission rates before adequate treatment and control measures can be instigated.

## Competing interests

The authors declare that they have no competing interests.

## Authors’ contributions

YRY, XLW and SKY participated in the data collection. YRY and MKE analysed the data. YRY, MKE, DPM, DJG, GMW and AGR were involved in drafting and revising the manuscript. All authors read and approved the final manuscript.

## Pre-publication history

The pre-publication history for this paper can be accessed here:

http://www.biomedcentral.com/1471-2458/12/1110/prepub
